# Impact of Perioperative HbA1c Levels on Functional Outcomes Following Open Carpal Tunnel Release: A Prospective Study

**DOI:** 10.7759/cureus.83615

**Published:** 2025-05-06

**Authors:** Erez Avisar, Eran Assaraf, Ahmad Essa, Kevin Zuo, Dror Lindner, Jonathan Persitz

**Affiliations:** 1 Hand and Upper Extremity Surgery Unit, Department of Orthopaedic Surgery, Yitzhak Shamir Medical Center, Tzrifin, ISR; 2 Department of Orthopaedic Surgery, Yitzhak Shamir Medical Center, Tzrifin, ISR; 3 Hand Program, Division of Plastic, Reconstructive and Aesthetic Surgery, University of Toronto, Toronto, CAN

**Keywords:** carpal tunnel release, carpal tunnel syndrome, glycated hemoglobin (hba1c), outcome analysis, patient-reported outcome measures

## Abstract

Purpose: The purpose of this prospective study was to evaluate the influence of perioperative glycemic control, as detected by glycosylated hemoglobin (HbA1c) levels, on clinical outcomes after open carpal tunnel release (CTR) surgery.

Methods: The demographic and clinical data of the study participants were prospectively collected prior to surgery and at one year postoperatively. Objective evaluations included grip and pinch strength, along with sensation testing over the index finger using Semmes-Weinstein monofilaments (SWMF). Subjective assessments, including pain intensity (measured by the visual analog scale (VAS)), the Disabilities of the Arm, Shoulder, and Hand (DASH) questionnaire, and the Mayo Wrist Score, were also recorded.

Results: The study included 50 patients, comprising 27 with type 2 diabetes mellitus (mean HbA1c: 7.49 ± 1.42) and 23 without diabetes. HbA1c levels were measured up to one month prior to surgery. No statistically significant differences were found in grip strength, pinch strength, or Semmes-Weinstein monofilament (SWMF) values pre- and postoperatively in both diabetic and non-diabetic groups. Pain intensity decreased in both groups at 12 months postoperatively, but the degree of pain improvement was not statistically different between groups. Both groups showed postoperative improvement in DASH and Mayo Wrist Scores, with no significant difference in the level of improvement between the groups. Pearson analysis showed no correlation between objective and subjective measures pre- and postoperatively and HbA1c levels.

Conclusions: Elevation of perioperative HbA1c levels in diabetic patients did not negatively affect surgical outcomes compared to non-diabetic patients. Both groups demonstrated significant functional improvements, with no notable differences in recovery or postoperative hand function.

## Introduction

Carpal tunnel syndrome (CTS) is the most common peripheral neuropathy, affecting approximately 4% of adults [1]. Carpal tunnel release (CTR) surgery is the standard treatment to improve sensation, relieve pain, and restore function [2]. Diabetes is a well-established risk factor for CTS, with a reported prevalence of 11%-21% among diabetic patients and up to 30% in those with diabetic peripheral neuropathy [3,4]. The pathophysiology likely involves endoneurial hypoxia and accumulation of advanced glycation end-products, contributing to nerve compression and collagen stiffening [5-7].

Glycosylated hemoglobin (HbA1c) is a biomarker reflecting average blood glucose levels over the preceding 2-3 months [8], and elevated levels are linked to diabetes-related complications, including neuropathy and cardiovascular disease [9-12]. HbA1c levels above 7% have also been associated with higher rates of surgical site infections (SSI), delayed wound healing, and other perioperative complications in upper extremity surgery [13].

Several studies have examined the risk of complications following CTR in diabetic patients, primarily focusing on SSI [14-16]. For instance, Cunningham et al. reported increased SSI risk in patients with HbA1c levels above 7.8% [14], while other authors observed similar minor complication rates among diabetic patients undergoing CTR [14-16].

In contrast, the impact of diabetes, and specifically perioperative glycemic control, on functional outcomes following CTR remains less well understood, with existing studies yielding mixed and often contradictory results [3,5,17]. Several studies suggest that while diabetic patients may present with more severe nerve compression, their postoperative outcomes are comparable to those of non-diabetic patients [3,17,18]. However, a key limitation of these investigations is the absence of stratification based on HbA1c levels or perioperative glycemic control.

The objective of this prospective study was to assess the impact of perioperative glycemic control, as reflected by HbA1c levels, on both clinical and patient-reported outcomes following open CTR surgery. The study specifically examined whether elevated HbA1c levels are associated with poorer postoperative recovery, including deficits in strength, sensation, and symptom relief.

## Materials and methods

Study design and population

This was a prospective observational study conducted at a tertiary referral center. Patients diagnosed with CTS in 2020 and referred to the Hand and Upper Extremity Surgery Unit were evaluated by a senior hand surgeon (EA). The diagnosis was confirmed based on CTS-6 clinical criteria and supported by nerve conduction studies.

Inclusion criteria were adult patients (>18 years) with a confirmed diagnosis of CTS scheduled for open CTR. Exclusion criteria included the following: type 1 diabetes, polyneuropathy, cervical radiculopathy or stenosis, recent wrist trauma, systemic inflammatory diseases, pregnancy, anemia, chronic oral steroid use, local steroid injection within six months prior to surgery, and unwillingness to participate.

The study was approved by the Ethics Committee of Yitzhak Shamir Medical Center (approval number: 0079-20), and written informed consent was obtained from all participants.

Surgical procedure

All patients underwent open CTR performed by one of two board-certified hand surgeons using a standardized technique. Surgery was performed under local anesthesia (5 mL of 1% lidocaine and 3 mL of 2.5% bupivacaine) and tourniquet control. A standard longitudinal incision was used. No prophylactic antibiotics were administered preoperatively.

Outcome measures and follow-up

Preoperative and 12-month postoperative assessments were conducted by the treating hand surgeons. Demographic data and comorbidities were collected preoperatively. Objective outcome measures included the following: grip strength (measured using Jamar dynamometer (TEC, Clifton, NJ)), pinch strength (measured using Jamar pinch gauge (Sammons Preston Inc., Bolingbrook, IL)), and fine sensation (tested using Semmes-Weinstein monofilaments (North Coast Medical, Inc., Morgan Hill, CA) on the index finger). Subjective outcome measures included pain intensity using a visual analog scale (VAS), function using the Disabilities of the Arm, Shoulder, and Hand (DASH) questionnaire, and overall wrist function using the Mayo Wrist Score. HbA1c levels were obtained within one month prior to surgery and at 12 months postoperatively in diabetic patients.

Statistical analysis

Data were analyzed using SPSS software (IBM Corp., Armonk, NY). Continuous variables were compared using independent t-tests, and categorical variables were analyzed using χ² or Fisher's exact tests, as appropriate. Within-group changes from pre- to postoperative assessments were analyzed using paired t-tests. Pearson correlation coefficients were used to assess the relationship between HbA1c and both objective and subjective outcomes in the diabetic group. Statistical significance was set at p < 0.05.

## Results

During the study period, 73 patients were indicated for open CTR surgery, and 50 were included in the study. This group included 27 patients with diabetes mellitus type 2 with a mean HbA1c value of 7.49 ± 1.42; the remaining 23 patients had normal HbA1c and no diagnosis of diabetes mellitus (Table 1).

**Table 1:** Demographic and clinical characterizations of the patients BMI: body mass index, R: right, L: left, HbA1c: glycosylated hemoglobin

Demographic factor	Diabetes (n = 27)	No diabetes (n = 23)	P-value
Sex (female/male)	22 (81.5%)/5 (18.5%)	16 (69.6%)/7 (30.4%)	0.325
Age (years)	59.4 ± 11.95	53.17 ± 14.39	0.061
BMI (kg/m²)	29.9 ± 4.10	28.11 ± 3.99	0.063
Operated hand (R/L)	15 (55.6%)/12 (44.4%)	7 (30.4%)/16 (69.6%)	0.075
Hand dominance (R/L)	4 (14.8%)/23 (85.2%)	0 (0%)/23 (100%)	0.076
Duration of symptoms (years)	3.41 ± 2.78	2.61 ± 2.80	0.159
Preoperative HbA1c	7.49 ± 1.42	N/A	0.159
Postoperative HbA1c (12 months)	7.14 ± 1.24	N/A	0.159

**Figure 1:**
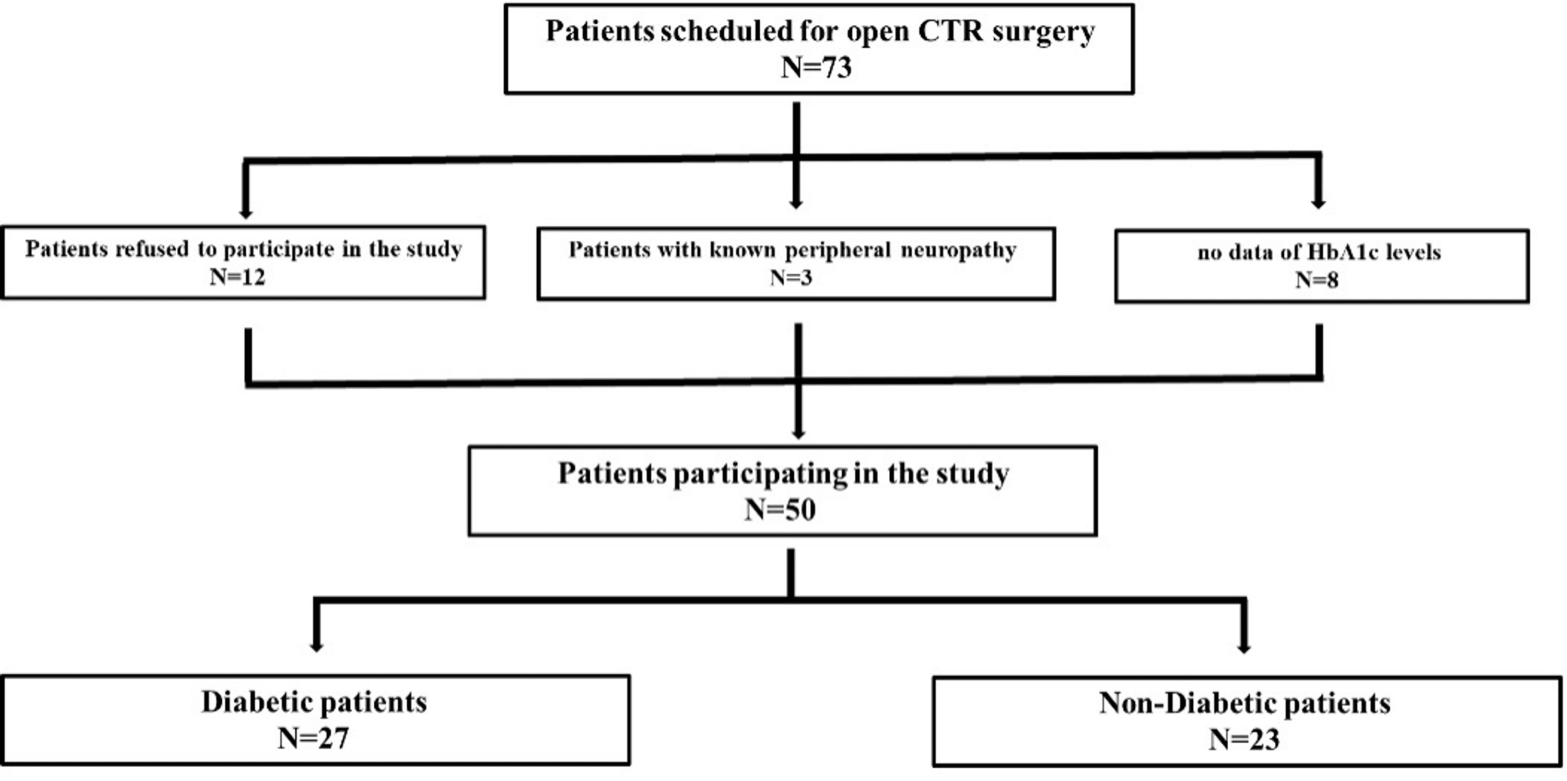
Flowchart describing the patients' recruitment process CTR: carpal tunnel release, HbA1c: glycosylated hemoglobin

HbA1c levels were measured up to one month prior to surgery. Of the remaining 23, 12 declined to participate in the study, three had known polyneuropathy, and eight diabetic patients were not tested for HbA1c levels and were therefore excluded from the study (Figure 1).

Demographics

Women were the predominant sex in both groups (22 (81.5%) in the diabetic group and 16 (69.6%) in the non-diabetic group). Age and BMI were similar between groups, with a mean age of 59.4 ± 11.95 years in the diabetic group and 53.17 ± 14.39 years in the non-diabetic group (P = 0.061), and BMI of 29.9 ± 4.10 kg/m² and 28.11 ± 3.99 kg/m², respectively (P = 0.063). The left hand was operated on more frequently in the diabetic group, while the right hand was more commonly operated on in the non-diabetic group (56% versus 70%, P = 0.075). Both groups had a right-hand dominance (85% and 100%). The duration of CTS was longer in the diabetic group, although not statistically significant (3.41 ± 2.78 versus 2.61 ± 2.80 years, P = 0.159). HbA1c levels in the diabetic group showed minimal change from pre- to 12 months post-operation (7.49 ± 1.42 versus 7.14 ± 1.24 mmol/mol, P = 0.159).

A subgroup analysis excluding diabetic patients with HbA1c < 7 (leaving those with a mean HbA1c of 8.3 preoperatively) demonstrated no change in the statistical significance of functional or subjective outcomes.

Functional outcome

All 50 patients completed both subjective and objective outcome measures. Functional measurements were compared before and 12 months after surgery in both diabetic and non-diabetic groups. The diabetic group showed mild grip strength improvement (17.59 ± 11.89 kg to 18.67 ± 9.97 kg), while no change was observed in the non-diabetic group (22.52 ± 7.72 kg to 22.70 ± 10.63 kg). Key pinch measurement showed no significant pre- to postoperative differences. SWMF measurements improved postoperatively in the diabetic group, with significant preoperative differences in the second finger compared to non-diabetic controls (0.87 ± 0.82 versus 0.18 ± 0.16, P < 0.0001) (Table 2).

**Table 2:** Objective measurements of hand function before and 12 months after open carpal tunnel release surgery

Measurements	Diabetes (n = 27) (54%)	No diabetes (n = 23) (46%)	P-value
Grip (kg)	Preoperative: 17.59 ± 11.89	Preoperative: 22.52 ± 7.72	0.085
	Postoperative: 18.67 ± 9.97	Postoperative: 22.70 ± 10.63	0.173
Pinch (kg)	Preoperative: 4.57 ± 2.96	Preoperative: 4.88 ± 2.50	0.692
	Postoperative: 4.74 ± 2.66	Postoperative: 4.67 ± 2.51	0.924
Semmes-Weinstein monofilament (index finger)	Preoperative: 0.87 ± 0.82	Preoperative: 0.18 ± 0.16	<0.001
Semmes-Weinstein monofilament (index finger)	Postoperative: 0.19 ± 0.38	Postoperative: 0.31 ± 0.55	0.972

Patient-reported outcomes

Pain VAS scores indicated high pain intensity preoperatively, which significantly decreased 12 months postoperatively in both groups (6.30 ± 2.98 to 2.35 ± 2.27, P < 0.001, in the non-diabetic group; 6.65 ± 2.62 to 2.15 ± 2.84, P < 0.001, in the diabetic group). Both groups showed improvement in DASH scores in the perioperative period (51.35 ± 17.55 to 27.58 ± 18.43, P < 0.001, in the non-diabetic group; 52.51 ± 15.18 to 24.09 ± 21.05, P < 0.001, in the diabetic group), with no significant difference between the groups. Similarly, improvement was observed in Mayo Wrist Scores in both groups ((preoperative: 50.56 ± 16.54 versus 50.22 ± 18.25, P = 0.946; postoperative: 76.30 ± 21.73 versus 80.65 ± 16.19, P = 0.432), P < 0.001 pre- versus post-operation), with no significant differences between groups (Table 3).

**Table 3:** Subjective measurements of hand function before and 12 months after open carpal tunnel release surgery VAS: visual analog scale, DASH: Disabilities of the Arm, Shoulder, and Hand

Measurements	Diabetes (n = 27) (54%)	No diabetes (n = 23) (46%)	P-value
Pain VAS	Preoperative: 6.65 ± 2.62	Preoperative: 6.30 ± 2.98	0.666
	Postoperative: 2.15 ± 2.84	Postoperative: 2.35 ± 2.27	0.787
DASH	Preoperative: 52.51 ± 15.18	Preoperative: 51.35 ± 17.54	0.803
	Postoperative: 24.09 ± 21.05	Postoperative: 27.58 ± 18.43	0.539
Mayo Wrist Score	Preoperative: 50.56 ± 16.54	Preoperative: 50.22 ± 18.25	0.946
	Postoperative: 76.30 ± 21.73	Postoperative: 80.65 ± 16.19	0.432

A Pearson correlation analysis was performed to assess the relationship between HbA1c values at 12 months postoperatively and the subjective and objective measures evaluated preoperatively and 12 months postoperatively in diabetic patients. Apart from a significant correlation between HbA1c and preoperative Mayo Wrist Scores (two-tailed t-test, P = 0.032), no other significant correlations were found between HbA1c and any other measures before or after surgery (Table 4 and Table 5). Notably, even after excluding diabetic patients with HbA1c levels less than 7 (with the remaining group with average HbA1c of 8.3 preoperatively and 8.1 at one-year follow-up), the statistical outcomes remained unchanged across all parameters.

**Table 4:** Pearson correlation analysis between HBA1c post-operation and objective measurements of hand function before and 12 months after open carpal tunnel release surgery in the diabetic group HBA1c: glycosylated hemoglobin

Measurements	Pearson correlation	Significance (two-tailed)
Grip (kg)	Preoperative: 0.342	0.081
	Postoperative: 0.079	0.696
Key pinch (kg)	Preoperative: 0.065	0.749
	Postoperative: -0.026	0.9
Semmes-Weinstein monofilament (index finger)	Preoperative: -0.114	0.57
	Postoperative: 0.157	0.434

**Table 5:** Pearson correlation analysis between HBA1c post-operation and subjective measurements of hand function before and 12 months after open carpal tunnel release surgery in the diabetic group HBA1c: glycosylated hemoglobin, VAS: visual analog scale, DASH: Disabilities of the Arm, Shoulder, and Hand

Measurements	Diabetes (n = 27) (54%)	No diabetes (n = 23) (46%)	P-value
Pain VAS	Preoperative: 6.65 ± 2.62	Preoperative: 6.30 ± 2.98	0.666
	Postoperative: 2.15 ± 2.84	Postoperative: 2.35 ± 2.27	0.787
DASH score	Preoperative: 52.51 ± 15.18	Preoperative: 51.35 ± 17.54	0.803
	Postoperative: 24.09 ± 21.05	Postoperative: 27.58 ± 18.43	0.539
Mayo Wrist Score	Preoperative: 50.56 ± 16.54	Preoperative: 50.22 ± 18.25	0.946
	Postoperative: 76.30 ± 21.73	Postoperative: 80.65 ± 16.19	0.432

## Discussion

Given the well-established association between elevated HbA1c and impaired wound healing, several studies have proposed a threshold of 7%-8% for increased SSI risk in diabetic patients undergoing upper extremity surgery [14,15,19,20]. However, to date, no studies have directly evaluated whether perioperative glycemic control, as measured by HbA1c, influences long-term functional recovery or patient-reported outcomes following CTR.

Some authors have speculated that decompression of the median nerve may be less effective in patients with uncontrolled diabetes due to underlying microvascular damage caused by chronic hyperglycemia [3,21]. The current study does not support this hypothesis. Our findings show that diabetic patients with elevated perioperative HbA1c levels achieved postoperative outcomes comparable to those of non-diabetic patients across all measured domains, including grip and pinch strength, Semmes-Weinstein monofilament (SWMF) testing, pain scores, DASH scores, and Mayo Wrist Scores.

Importantly, while prior studies have evaluated functional outcomes in diabetic versus non-diabetic patients, few have incorporated HbA1c as a continuous or stratified variable. This study adds to the limited body of evidence by prospectively assessing patients with varying degrees of glycemic control. Despite the inclusion of diabetic patients with HbA1c levels above 8%, no statistically significant differences were observed in either objective or subjective recovery metrics. This suggests that moderate elevations in HbA1c may not independently compromise functional outcomes in appropriately selected surgical candidates.

Our results are consistent with prior research showing similar functional improvements in diabetic and non-diabetic patients following CTR. Zyluk and Puchalski reported comparable gains in strength and sensation in a mixed cohort of 386 patients [22], while Thomsen et al. found no significant differences in long-term motor and sensory function at five years postoperatively between matched diabetic and non-diabetic groups [17]. A population-based analysis by Zimmerman et al. of over 10,000 hands in the Swedish National Quality Registry similarly demonstrated that, although diabetic patients had higher baseline DASH scores, their absolute improvement was comparable to non-diabetic patients [23].

An unexpected finding in the non-diabetic cohort was a slight, non-significant increase in SWMF values postoperatively, suggesting a potential decline in fine sensation. However, the clinical relevance of this trend is uncertain given the small sample size and large postoperative variability. This may reflect measurement inconsistency rather than true sensory worsening, but further investigation in larger cohorts is warranted.

To further evaluate the effect of poor glycemic control, we performed a subgroup analysis excluding diabetic patients with HbA1c < 7. Among this subgroup, representing patients with suboptimal control (mean HbA1c: 8.3%), no differences were found compared to the full diabetic cohort. This reinforces the notion that elevated HbA1c levels, within the ranges observed in our study, may not preclude meaningful symptom relief or functional recovery following CTR.

Our study has several limitations. First, although prospectively designed, the sample size was determined by the number of eligible patients during the study period and was not based on an a priori power analysis. As a result, the study may be underpowered to detect subtle or moderate differences between groups. Second, outcomes were assessed at a single one-year postoperative time point. While this captures longer-term recovery, it may not reflect potential differences in the early postoperative phase, such as pain or functional variability in the first few months. Conducting the study at a single center with a uniform surgical technique improves comparability, although it may limit generalizability. To address this, we ensured standardized surgical and postoperative protocols, reducing variability within our cohort. Future multicenter studies incorporating varied surgical approaches and perioperative protocols would help validate our findings across different settings. Additionally, the study did not stratify patients based on baseline CTS severity, which may have influenced the variability in postoperative recovery and limited the ability to adjust for disease stage as a potential confounder. While our study is limited to patients already selected for surgical treatment and does not explore the full spectrum of glycemic control in the general diabetic population, it provides valuable insight into the safety and functional recovery in diabetic patients undergoing CTR.

## Conclusions

Elevation of perioperative HbA1c levels in diabetic patients did not negatively affect surgical outcomes compared to non-diabetic patients. Both groups demonstrated significant functional improvements, with no notable differences in recovery or postoperative hand function. However, larger prospective studies are needed to further evaluate the long-term impact of glycemic control on surgical outcomes and confirm these findings across diverse patient populations.
